# Sex‐dependent improvement in traumatic brain injury outcomes after liposomal delivery of dexamethasone in mice

**DOI:** 10.1002/btm2.10647

**Published:** 2024-02-04

**Authors:** Gherardo Baudo, Hannah Flinn, Morgan Holcomb, Anjana Tiwari, Sirena Soriano, Francesca Taraballi, Biana Godin, Assaf Zinger, Sonia Villapol

**Affiliations:** ^1^ Department of Nanomedicine Houston Methodist Research Institute Houston Texas USA; ^2^ Department of Neurosurgery and Center for Neuroregeneration Houston Methodist Research Institute Houston Texas USA; ^3^ Department of Orthopedics and Sports Medicine and Center for Musculoskeletal Regeneration Houston Methodist Hospital Houston Texas USA; ^4^ Department of Obstetrics and Gynecology Houston Methodist Research Institute Houston Texas USA; ^5^ Department of Obstetrics and Gynecology Weill Cornell Medicine College (WCMC) New York New York USA; ^6^ Department of Biomedical Engineering Texas A&M University College Station Texas USA; ^7^ Department of Cardiovascular Sciences Houston Methodist Research Institute Houston Texas USA; ^8^ Department of Chemical Engineering Technion−Israel Institute of Technology Haifa Israel; ^9^ Department of Neuroscience in Neurological Surgery Weill Cornell Medicine College (WCMC) New York New York USA

**Keywords:** brain injury, cytokines, drug delivery, glucocorticoid, inflammation, sex differences

## Abstract

Traumatic brain injury (TBI) can have long‐lasting physical, emotional, and cognitive consequences due to the neurodegeneration caused by its robust inflammatory response. Despite advances in rehabilitation care, effective neuroprotective treatments for TBI patients are lacking. Furthermore, current drug delivery methods for TBI treatment are inefficient in targeting inflamed brain areas. To address this issue, we have developed a liposomal nanocarrier (Lipo) encapsulating dexamethasone (Dex), an agonist for the glucocorticoid receptor utilized to alleviate inflammation and swelling in various conditions. In vitro studies show that Lipo‐Dex were well tolerated in human and murine neural cells. Lipo‐Dex showed significant suppression of inflammatory cytokines, IL‐6 and TNF‐α, release after induction of neural inflammation with lipopolysaccharide. Further, the Lipo‐Dex were administered to young adult male and female C57BL/6 mice immediately after controlled cortical impact injury (a TBI model). Our findings demonstrate that Lipo‐Dex can selectively target the injured brain, thereby reducing lesion volume, cell death, astrogliosis, the release of pro‐inflammatory cytokines, and microglial activation compared to Lipo‐treated mice in a sex‐dependent manner, showing a major impact only in male mice. This highlights the importance of considering sex as a crucial variable in developing and evaluating new nano‐therapies for brain injury. These results suggest that Lipo‐Dex administration may effectively treat acute TBI.

**Translational Impact Statement**

This research introduces a novel approach for delivering dexamethasone enclosed within liposomal nanoparticles to the brain following a brain injury in mice. The findings illustrate a significant decrease in both brain damage and the associated inflammatory response, all without inducing any toxic effects. This innovative brain‐targeted delivery method holds promising potential for enhancing the efficacy of trauma treatments.

## 1 INTRODUCTION

Traumatic brain injury (TBI) leads to three million emergency room visits annually.^1^ TBI promotes robust neuroinflammation by activating microglia and macrophages that release pro‐inflammatory cytokines and chemokines. This process attracts leukocytes, neutrophils, T cells, monocytes, and infiltrating macrophages, which increase neurotoxicity and neuronal death.^2^ The invasion of immune cells contributes to a larger lesion size and a slower recovery, exacerbating neurotoxicity and neuronal death.^3^, ^4^ However, drug delivery to the brain has proven challenging, making pharmacotherapy for TBI ineffective. Systemic drug administration, with sufficient concentration to reach the injured brain, may result in toxicity to other organs. TBI has the potential to disrupt the blood–brain barrier (BBB), which typically serves as a protective barrier between the brain and the rest of the body, controlling the flow of substances into and out of the brain. While this consequence from TBI can facilitate drug delivery to the brain, it can also allow harmful substances to enter, potentially causing further damage. Even if a drug can cross the BBB, delivering it to the specific brain region in need may be difficult. The brain is highly compartmentalized, and the drug may not be able to reach certain areas due to their location or the nature of the injury. This could potentially increase the risk of a secondary injury and disrupt the body's natural healing processes.

Dexamethasone (Dex) is a glucocorticoid employed as an anti‐inflammatory agent in diverse medical conditions, including injuries to the central nervous system (CNS).^5^ It has been used to treat brain tumors, critical brain illness, stroke, and COVID‐19.^6^, ^7^, ^8^ Despite its efficacy, Dex cannot accumulate in the brain due to its active efflux transport by P‐glycoprotein.^9^ In addition, systemic administration of Dex is associated with several side effects, including glucose intolerance, immunosuppression, and neuropsychiatric problems.^9^, ^10^, ^11^ Consequently, despite a potent anti‐inflammatory action, the long‐term clinical outcomes of TBI patients treated with Dex are not significantly different or worse than those treated with a placebo.^12^, ^13^ Thus, an efficient delivery method is needed to control the therapeutic concentration of Dex at the target site and limit its distribution after TBI. Glucocorticoids are the main physiological hormones with anti‐inflammatory effects and exhibit the sexual dimorphism that occurs in inflammatory conditions.^14^ While estrogens can promote inflammation via Akt–mTOR pathway,^15^ male steroid hormones generally suppress immune function^16^ through inhibition of NF‐κB and COX‐2. These differences in hormones between males and females demonstrate that the effect of Dex on the inflammatory state can be sex dependent.

Efficient drug delivery to the brain for treating neuropathological conditions remains a significant challenge. Liposomes (Lipo) are clinically used nanoparticles composed of phospholipid bilayers surrounding an aqueous core,^17^ which can improve site‐specific drug delivery and shelf‐life while avoiding adverse effects.^18^, ^19^, ^20^ Lipo show promise in enabling targeted delivery of drugs to the brain by circumventing efflux transporters^21^ or targeting specific cell types.^21^, ^22^ The compromised BBB following TBI may provide an opportunity for enhanced delivery of Lipo to damaged cortical regions. Although Dex encapsulated in Lipo were used before in animal models of arthritis and in vitro,^23^, ^24^ to the best of our knowledge, and up to this date, their efficacy in a TBI model was never assessed while evaluating the sex effect on the therapeutic outcomes. Direct delivery of anti‐inflammatory agents to the affected brain areas using Lipo can potentially mitigate the harmful effects of neuroinflammation and promote recovery after TBI, while minimizing off‐target effects and drug toxicity compared to other delivery methods. Our recent work shows that Lipo can be efficiently delivered to the brain after TBI.^25^ More importantly, this study also demonstrated that empty Lipo possess therapeutic benefits, as evidenced by reduced brain lesions. Nonetheless, additional research is necessary to optimize Lipo‐based therapies for TBI and other neuropathological conditions, focusing on their lipid backbone and the therapeutic cargo they can deliver.

Here, we aimed to investigate the therapeutic potential of encapsulating Dex in Lipo (Lipo‐Dex) with the same composition as in our previous work.^25^ In this study, we explored the capability of Lipo‐Dex to alleviate inflammation both in vitro and in vivo. The administration of Lipo‐Dex diminished neuroinflammation following TBI in mice, displaying a sex‐dependent pattern. This research underscores the potential of nanotechnology in advancing more precise and effective treatments for brain injuries.

## 2 MATERIALS AND METHODS

### 2.1 Lipo‐Dex design and purification

**FIGURE 1:**
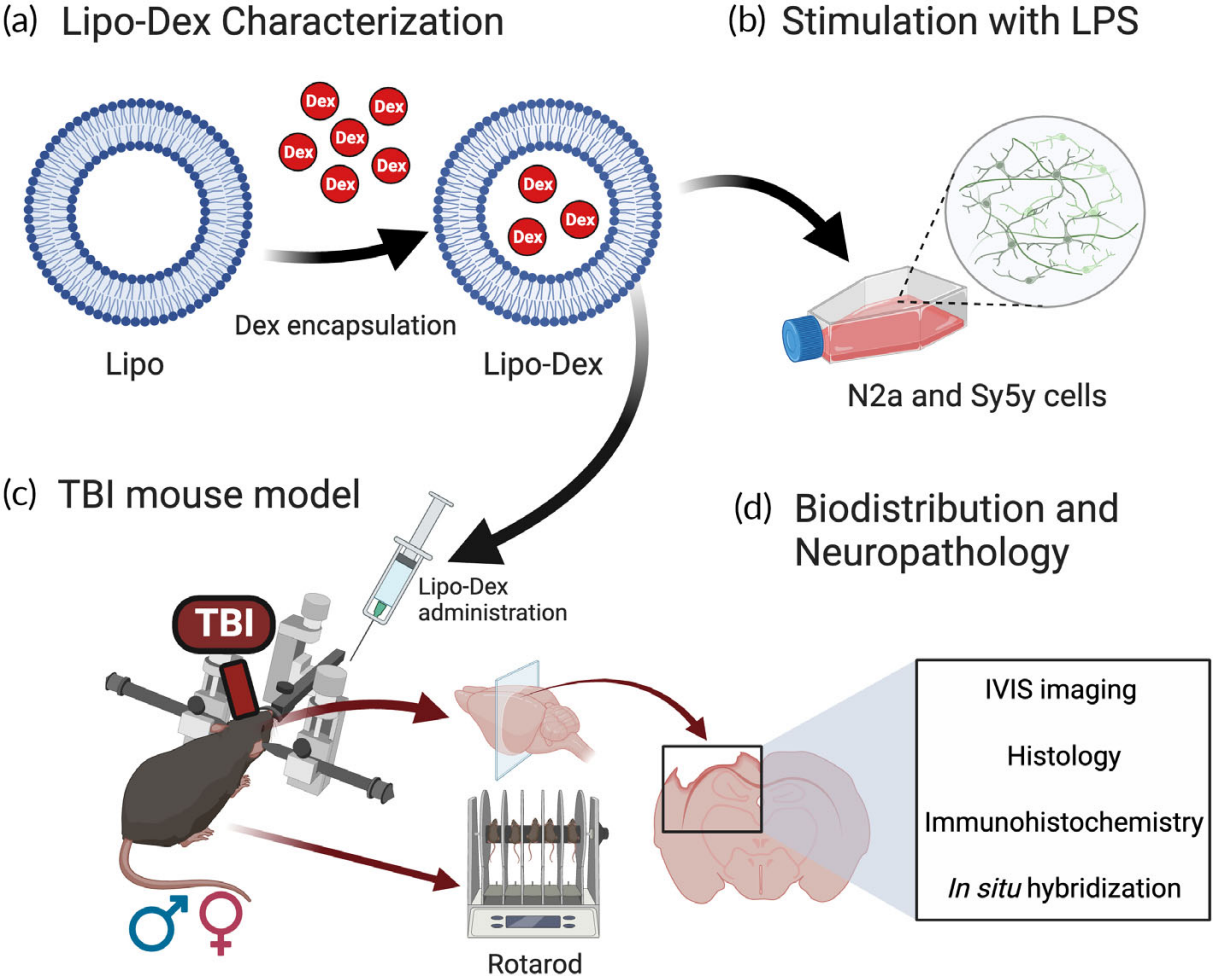
Schematic of Lipo‐Dex fabrication, characterization, and in vivo and ex vivo experiments. (a) Dex encapsulation was performed in vitro and after fabrication, Lipo‐Dex were characterized for their physicochemical properties and Dex release measurements. (b) Neuronal cells (N2a and Sy5y) were stimulated with LPS and inflammatory cytokines were measured. (c) Lipo‐Dex were tested in vivo using a TBI mouse model, and (d) the therapeutic effect was evaluated using in vivo imaging system (IVIS), histological, immunohistochemical, and in situ hybridization techniques while assessing the mice sex effect on the therapeutic outcomes. TBI, traumatic brain injury.

To encapsulate Dex in Lipo, we followed our previously published protocol^25^ (Figure 1a). Both Lipo and Lipo‐Dex were prepared using the same extrusion parameters, with the exception of adding Dex during the lipids thin‐film rehydration. The systems were designed using a 4:3:3 molar ratio of dipalmitoylphosphatidylcholine (DPPC), dioleoylphosphatidylcholine (DOPC), and cholesterol (Avanti Polar Lipids, Inc., Alabaster, USA) at a lipid concentration of 50 mM. Next, the lipids were dissolved in chloroform (Sigma Aldrich, Missouri, USA) at a final concentration of 20 mg/mL, mixed, and sonicated for 5 min at 45°C. The chloroform was then evaporated using a rotary evaporator (BUCHI Labortechnik AG, Flawil, Switzerland) at 45°C, 280 rpm for 30 min, and the lipid film obtained was hydrated for 30 min at 50°C and 280 rpm with either 2 mL phosphate‐buffered saline (PBS, Fisher Scientific, Hampton, USA) for Lipo or 2 mL of 25 mg/mL dexamethasone (Sigma‐Aldrich, St. Louis, USA) for Lipo‐Dex. Subsequently, Lipo and Lipo‐Dex were extruded at 50°C through 80–200 nm polycarbonate membranes (Whatman, Buckinghamshire, UK). For biodistribution studies, 0.1 mg of Cy5.5‐DSPE (Avanti Polar Lipids, Inc., Alabaster, USA) were dissolved in chloroform (100 μL of 1 mg/mL) together with other lipids to fluorescently label both Lipo groups. Lipo‐Dex were dialyzed for 19 h in PBS using 1000 kDa float A‐Lyzer (Spectrum™ Labs, Massachusetts, USA), with the buffer exchanged after 1 and 3 h. Subsequently, the samples were sterilized using a 0.22 μm PVDF filter.

### 2.2 Lipo and Lipo‐Dex characterization

The physical and chemical properties of both systems were evaluated as we previously described.^26^ Briefly, liposomal systems were characterized using dynamic light scattering (DLS) (Malvern Instruments, Worcestershire, UK) to determine their polydispersity index (PDI), diameter, and zeta potential (ZP). Three measurements, consisting of 10 runs each, were taken to determine the size and PDI of the liposomes, while ZP was measured using three measurements, consisting of 15 runs each. To assess the effect of serum on physicochemical parameters of Lipo‐Dex, the system was mixed in 10% FBS prior to assessing PDI, diameter, and ZP. The fluorescence of Lipo‐Dex labeled with Cy5.5 lipid was assessed at 678 nm excitation and 707 nm emission using the FLUOstar Omega microplate reader (BMG, Labtech Ortenberg, Germany).

### 2.3 Encapsulation and drug release studies

The efficiency of Dex encapsulation in Lipo and its subsequent release was evaluated with high‐performance liquid chromatography (HPLC) using the 1260 Infinity II LC System (Agilent Technologies, California, USA). A Luna® 5 μm C18(2) 100 Å, LC Column 250 x 4.6 mm (Phenomenex, California, United States) was used. The samples were mixed with PBS and acetonitrile (ACN) at a ratio of 1:6:3 (v/v/v) and sonicated, followed by centrifugation and transfer to HPLC vials. The tubes were warmed for 10 min at 40°C and then sonicated for 10 min at 40°C. Subsequently, the samples were transferred inside the centrifugal devices (Pall Corporation, NY, USA) and centrifuged for 10 min, 17,000 rcf at 40°C. After the centrifugation, the samples were transferred into the HPLC vials; 70% of MilliQ water and 30% ACN were used under the isocratic mobile phase. Samples ran under a 1 mL min^−1^ flow and absorbance was measured at 254 nm. To measure mass loading of Dex in Lipo‐Dex, Dex concentration was assessed by HPLC, while the phospholipid concentration was measured by EnzyChrom™ Phospholipid Assay Kit (Fisher Scientific, USA, Cat. #50‐489‐287). The mass loading was calculated as mass of Dex divided by mass of lipids in the system. Dex release profile from Lipo was assessed by 1000 kDa Float‐A‐Lyzer dialysis in PBS under agitation at 37°C. The encapsulation efficiency of Dex was calculated as the ratio between Dex encapsulated after filtration and Dex encapsulated after extrusion measured by HPLC.

### 2.4 Cryo‐transmission electron microscopy

Cryo‐transmission electron microscopy (Cryo‐TEM) at the Baylor College of Medicine Cryo‐Electron Microscopy Core Facility (Houston, TX, USA) was used to analyze the structure of the Lipo and Lipo‐Dex particles. The Quantifoil R2/1, Cu 200 mesh Holey Carbon grids were pretreated with a 45 s air‐glow discharge to make the carbon surface hydrophilic. Quantifoil R2/1200Cu 4 nm thin carbon grids were glow discharged for 10 s to the efficacy of the added layer of continuous carbon with binding of the Lipo. Vitrification was performed using a Vitrobot Mark IV (FEI, Hillsboro, OR, USA) operated at 18°C and 100% humidity. Each grid contained 3 μL of Lipo or Lipo‐Dex samples that was blotted for 1–3 s before being submerged in liquid ethane. The frozen grids were then transferred into a JEOL 3200FS microscope (JEOL) outfitted with a Gatan K2 Summit 4kx4k direct detector (Gatan, Pleasanton, CA, USA) and a post column energy filter set to 30 eV. The microscope was aligned prior to imaging to prevent poor image quality from possible beam‐induced aberrations or astigmatism. Samples were imaged using a magnification of 15,000× and 30,000× with respective pixel sizes of 2.392 and 1.232 angstroms. Image exposure was limited to 1 s with an approximate dose rate of ≈20e−/Å2/s per image. The thickness of the phospholipid bilayer was measured for 10 Lipo and Lipo‐Dex using the Fiji software.

### 2.5 In vitro studies in neuronal cell culture

SH‐Sy5y (human cells derived from a female neuroblastoma patient) and N2a (Neuro‐2a, derived from neuroblastoma in mice) cell lines were obtained from Sigma (USA) and ATCC (USA), respectively. The cells were cultured with Dulbecco's modified Eagle's medium (DMEM, Thermo Fisher Scientific, Inc., Waltham, MA, USA) supplemented with 10% fetal bovine serum (FBS, Thermo Fisher Scientific) and 1% penicillin–streptomycin. In vitro biocompatibility of Lipo‐Dex was determined using WST‐1 assay. Cells were seeded at a density of 5000 cells per well in two 96‐well plates and allowed to incubate overnight. Subsequently, they were subjected to Lipo‐Dex treatment at concentrations of 0.001, 0.01, 0.1, 1, and 10 μM, followed by incubation for 24 and 48 h (*n* = 5). WST‐1 assay reagent was added after 24 and 48 h incubation for 4 h and absorbance was measured at 490 nm. To access the ability of Lipo‐Dex in preventing inflammation, in vitro, Sy5y and N2a cells were seeded on a 24‐well plate at 1 x 10^5^ cells per well 24 h prior to the experiments. The cells were pretreated for 1 h with Lipo‐Dex (at Dex concentrations of 1 μM and 5 μM) resuspended in a fresh media for 1 h and stimulated with LPS (5 μg/mL) for 24 h (Figure 1b). Cell culture media from each well was collected and stored at −20°C until analysis. Subsequent cytokine measurements interleukin‐6 (IL‐6) and tumor necrosis factor‐alpha (TNF‐α) were done using Milliplex magnetic bead panel (Millipore Sigma, MA, USA) according to the manufacturer's protocol. Standard curves and cytokine concentrations of the samples were generated with the Luminex 200 software.

### 2.6 Mice and TBI model

Twelve‐week‐old C57BL/6J mice of both male and female sexes, sourced from Jackson Laboratories in Bar Harbor, ME, USA, were accommodated in the animal facilities of the Houston Methodist Research Institute. All animal studies were approved by the Institutional Animal Care and Use Committee (IACUC) at the Houston Methodist Research Institute (IACUC: IS00004860). The mice had access to food and water ad libitum and were kept under a 12 h light and dark cycle. During surgical procedures, isoflurane was used to anesthetize all mice (3% for induction, for 1.5%–2% maintenance). An electromagnetically controlled cortical impact (CCI) injury tool (Impact One stereotaxic impactor, Leica Microsystems, Buffalo Grove, IL, USA) was used to perform a left sided moderate TBI at the primary motor and somatosensory cortices as previously described in our laboratory.^27^, ^28^, ^29^ The injury was made using a 3 mm diameter flat impact tip with the set parameters of an impact velocity of 3.25 m/s and a 1.5 mm impact depth. Direct impact was made 2 mm lateral and 2 mm posterior to the bregma to induce an adequate acute inflammatory response post‐injury (Figure 1c). Sham mice underwent all procedures, including anesthesia, but did not undergo a cortical impact. Following that, biodistribution and neuropathology studies were carried out (Figure 1d).

### 2.7 Biodistribution of Lipo‐Dex using IVIS imaging

Lipo‐Dex were labeled with Cy5.5‐DSPE and their fluorescence intensity was measured as described above. The labeled Lipo‐Dex (100 μL/per mouse; 3 mg Dex/kg) were then administered intravenously via the retro‐orbital route into male and female C57BL/6J mice 1 h after CCI injury (*n* = 6/group, 3 males and 3 females). Mice were euthanized 1 day post‐TBI and their organs (brain, heart, lungs, liver, spleen, and kidneys) and blood were collected and imaged ex vivo using in vivo imaging system (IVIS) to assess nanoparticle biodistribution. Serum separation was performed by centrifugation at 4000 rpm for 20 min at 4°C for inflammatory cytokine analysis. The IVIS imaging was conducted using the following acquisition parameters: Ex = 640 nm, Em = 720 nm, Epi‐illumination, Bin: (HR)4, FOV: 18.4, f2, 0.5 s. The Living Image software was used to analyze the data.

### 2.8 Cresyl violet staining and lesion volume measurements

The brain tissues were fixed in 4% paraformaldehyde overnight, followed by storage in a 30% sucrose solution at 4°C. Brains were then sectioned at 15 μm thickness from the coronal plane through the dorsal hippocampus using a cryostat (Epredia Cryostar NX50, Fisher Scientific, Waltham, MA, USA). Sections were then submerged in a cryoprotective antifreeze solution (30% glycerol, 30% ethylene glycol, 40% 0.01 M PBS) to maintain tissue integrity for storage at −20°C. A cresyl‐violet solution was prepared under a ventilated hood by combining 0.1% cresyl‐violet (Sigma‐Aldrich, St. Louis, MO, USA) with distilled water. The solution was then heated and mixed with a stir bar before being filtered. The prepared sections were mounted onto gelatin‐coated glass slides (SuperFrost Plus, ThermoFisher Scientific, IL, USA), rehydrated, and stained with the cresyl‐violet solution for 10 min. After staining, the slides were dehydrated sequentially with varied dilutions of ethanol and xylene before being covered with a xylene based Permount (ThermoFisher Scientific) mounting media and cover slipped. Slides were then imaged to assess the lesion of both groups. The lesion volume was calculated by dividing the volume of the lesion in the left hemisphere by the total volume of the ipsilateral right hemisphere, which was obtained in a similar manner by multiplying the combined areas of the ipsilateral hemispheres by the distance between sections.

### 2.9 Organs paraffin embedding and hematoxylin and eosin staining

The collected organs, including the heart, lungs, liver, spleen, and kidneys, were initially fixed in 4% paraformaldehyde for 48 h and then transitioned to 70% ethanol. These tissues underwent processing using a Shandon Exelsion ES Tissue Processor and were subsequently embedded in paraffin using the standard protocols recommended by the manufacturer. The organs were sectioned into 5 μm‐thick slices. Following this, the sections underwent two 30‐min dehydration steps in 95% ethanol, were soaked in xylene for 1 h at a temperature between 60 and 70°C, and then placed in paraffin for 12 h. After the dehydration process, the tissues were stained with hematoxylin solution for 6 h at a temperature range of 60–70°C. The stained tissues were rinsed under tap water until the excess stain was removed. Differentiation of the tissue was achieved by treating it with a solution of 10% acetic acid and 85% ethanol diluted in water, with this process being repeated twice for 2 min before a final rinse with tap water.

### 2.10 Immunofluorescence analysis and cell death assay

For immunostaining, brain sections were first washed with 0.5% PBS‐Triton X‐100 (PBS‐T) for 5 min before applying a 3% normal goat serum (NGS, #1000, Vector Laboratories, Burlingame, CA, USA) blocking solution for 1 h at room temperature (RT). Next, brain sections were incubated at 4°C overnight in a primary solution made from blocking solution (PBS‐T and 3% NGS) and the following primary antibodies: anti‐rabbit Iba‐1 (1:500, Wako) for activated microglia/macrophages, anti‐rabbit glial fibrillary acidic protein (GFAP) (1:1000, Dako), and anti‐mouse S100β (1:200, Sigma). The following day, sections were washed with PBS‐T before applying the corresponding conjugated IgG secondary antibodies: anti‐rabbit or anti‐mouse Alexa Fluor 568 and anti‐rat Alexa Fluor 488 (all 1:1000, Thermo Fisher Scientific). Samples were incubated at RT for 2 h. Lastly, cell nuclei were counterstained with DAPI solution diluted in PBS (1:50,000, Sigma‐Aldrich), and slides were cover slipped using Tris Buffer mounting medium (Electron Microscopy Sections, Hatfield, PA, USA). To assess cell death, brain sections were separately processed for DNA strand breakage using Terminal deoxynucleotidyl transferase dUTP nick end labeling (TUNEL) from Fluorescence In Situ Cell Death Detection kit (Roche Diagnostic, Indianapolis, IN, USA) according to the manufacturer's instructions. For double immunostaining, brain sections were incubated with primary antibody against monoclonal anti‐mouse NeuN (1:200, Sigma‐Aldrich) overnight at 4°C. Subsequently, Alexa Fluor 568‐conjugated goat anti‐mouse IgG (1:1000, Invitrogen) was applied for 1 h at RT. The brain sections were then rinsed with PBS three times for 5 min each and immersed in PBS with DAPI solution. After this, the sections were rinsed with distilled water and cover‐slipped using Fluoro‐Gel with Tris Buffer mounting medium. TUNEL‐positive nuclei were quantified in five cortical regions (20× magnification) across three to five coronal sections for each animal (*n* = 5/group).

### 2.11 Fluorescent in situ hybridization with immunohistochemical labeling

Brain sections in the coronal plane were affixed to glass slides coated with gelatin (Superfrost Plus, Thermo Fisher Scientific) and then stored at a temperature of −80°C. Subsequently, fluorescent in situ hybridization (FISH) was carried out using RNAscope® Technology 2.0 Red Fluorescent kit (Advanced Cell Diagnostics (ACD), Hayward, CA, USA) as we previously described.^27^, ^28^ Brain tissue sections were first dehydrated by 50%, 70%, and 100% ethanol gradually for 5 min; pretreatment 1 was applied for 10 min, then they were boiled for 5 min with pretreatment 2 solution (citrate buffer), then incubated with pretreatment 3 solution (protease buffer) for 30 min before hybridization. Sections were incubated at 40°C for 2 h with the following mouse target probes: *Mus musculus* Il1b mRNA (Cat. No. 316891, ACD) and separately with *Mus musculus* TNF‐α (Cat. No. 311081, ACD). In addition, the negative (Cat. No. 310043, ACD) and positive (Cat. No. 313911, ACD) control probes were applied and allowed to hybridize for 2 h at 40°C. The amplification step was performed according to manufacturer's directions. Slides were washed three times with PBST and blocked with PBST and 5% normal goat serum for 1 h. Immunofluorescent labeling for microglia and macrophage cells was performed by incubating samples with a polyclonal anti‐rabbit Iba‐1 (1:500, 019‐9741, Wako Chemicals, Richmond, VA, USA) antibody overnight at 4°C. A secondary Alexa Fluor 488‐conjugated goat anti‐rabbit (1:1.000, Invitrogen, Carlsbad, CA, USA) was applied for 2 h at RT. Sections were rinsed with PBS three times in intervals of 5 min and then incubated DAPI solution diluted in PBS for 5 min to counterstained nuclei. The mounted sections were washed with distilled water and cover slipped with Fluoro‐Gel and a Tris Buffer mounting medium (Electron Microscopy Sciences).

### 2.12 Quantitative analysis of immunohistochemistry and confocal microscopy imaging

Images were captured using a Nikon motorized fluorescence microscope (Eclipse Ni‐U, Melville, NY, USA) equipped with a pco.edge sCMOS camera (4.2LT USB3) and analyzed utilizing NIS‐Elements software. For quantitative analysis of immunolabeled sections, we implemented unbiased standardized sampling techniques to measure tissue areas corresponding to the cortex and corpus callosum showing positive immunoreactivity. To quantify the number of Iba‐1 positive cells, an average of five single plane sections from the lesion epicenter (−1.34 to –2.30 mm from bregma) were analyzed blind for each animal for each brain region. Within each brain region, every Iba‐1, S100β, and mRNA‐positive cells in each of the five fields in the cortex (×20, 151.894 mm^2^) around the impact area were analyzed. For proportional area measurements, the magnitude of the individual reaction for microglial and astroglial cells was reported as the proportional area of tissue occupied by immunohistochemical stained cellular profiles within the cortex, hippocampus, and corpus callosum regions. Data are shown as the percentage of Iba‐1 or GFAP‐positive immunoreactivity per the total area occupied on the field studied. We conducted a quantitative assessment of immunoreactive areas for Iba‐1 and GFAP in 15 cortical, hippocampus, and corpus callosum regions taken at the level of the impact site. Images were transferred to ImageJ64 software (NIH, Bethesda, MD, USA), for inversion, thresholding, and densitometric analysis. The threshold function is used to set a black and white threshold corresponding to the imaged field, with the averaged background subtracted out. Once a threshold is set, the “Analyze Particles” function can be used to sum up the total area of positive staining, and to calculate the fraction of the total area. Co‐localization of IL1‐β and TNF‐α expression with microglia/macrophage markers was evaluated with *z*‐stack acquisitions using the confocal microscope Leica DMi8.

### 2.13 Rotarod test

To evaluate motor performance and coordination in the mice, we used a rotating rod apparatus (Ugo Basile Harvard Apparatus, PA, USA). The mice were positioned on a rotating rod, with the speed incrementally escalating from 4 to 40 revolutions per minute (rpm). We measured the time it took for the mice to fall from the rod, recording this as the latency to fall in seconds. The mice underwent training 2 days prior to the CCI injury, and their initial performance was established by testing them on the day of the surgery. A subsequent test was conducted 1 day after the CCI injury (*n* = 5 mice/group).

### 2.14 Statistical analysis

Statistical probabilities for size, PDI, ZP, and NPs concentration during formulation steps among different NPs formulations were determined using an unpaired *t*‐test. For biodistribution studies, a one‐way ANOVA followed by a Tukey's multiple comparison test was used to determine statistical probability. Analysis of the rotarod test and histochemical/immunofluorescence utilized a one‐way ANOVA to compare the time after injury and sex as the independent variables. A post hoc test with Tukey's multiple comparisons test  was then applied. All data in the study were presented as mean ± SEM. *p < 0.05, **p < 0.01, ***p < 0.001, and ****p < 0.0001 were considered statistically significant. GraphPad Prism 8 Software (GraphPad, San Diego, CA, USA) was used for statistical analysis.

## 3 RESULTS

### 3.1 Fabrication, characterization, and stability assessment of Lipo and Lipo‐Dex in storage

**FIGURE 2:**
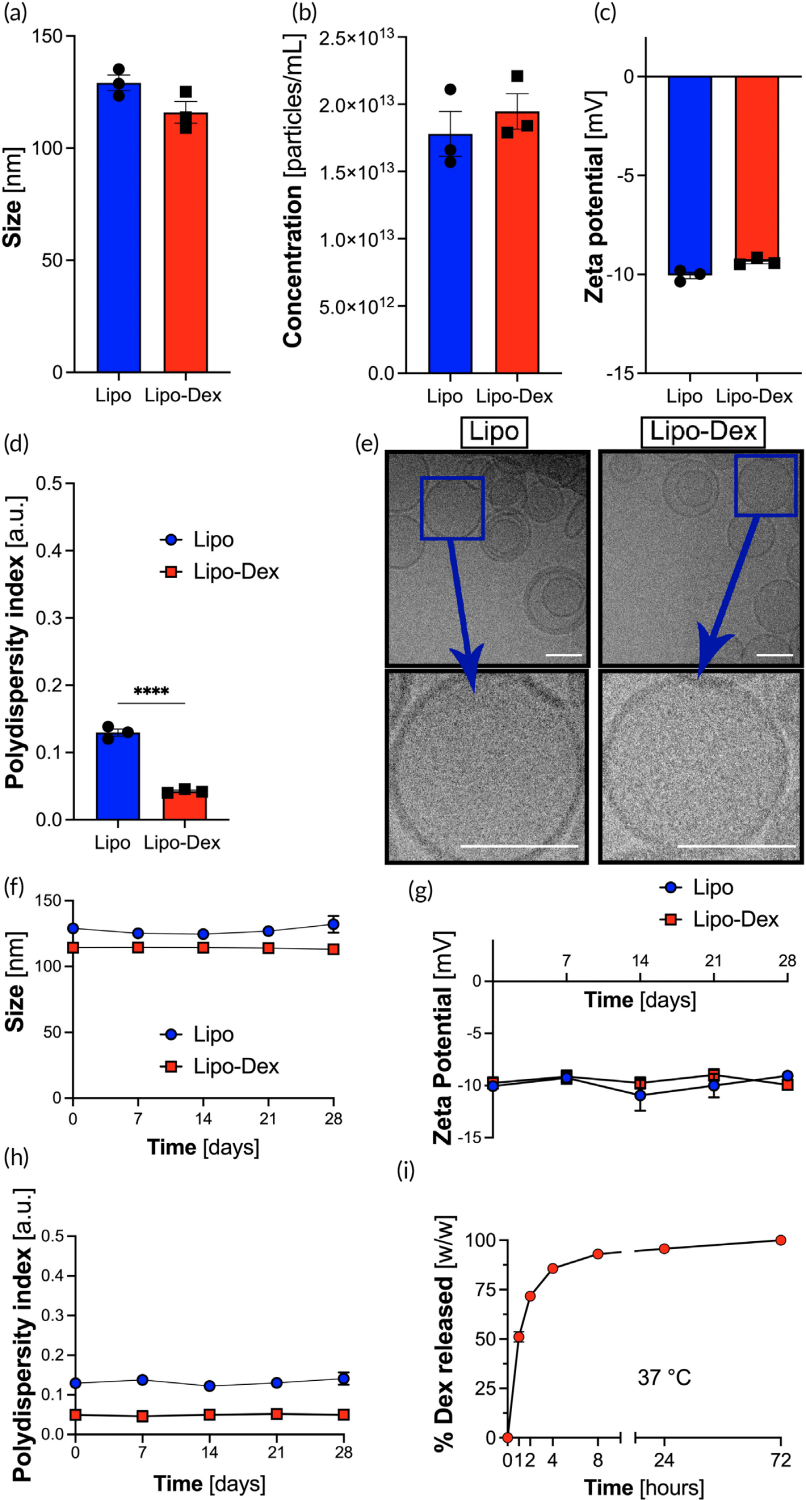
Fabrication, characterization, and stability assessment of Lipo and Lipo‐Dex in storage and body temperatures. Lipo and Lipo‐Dex were characterized for their physicochemical properties. No significant changes in (a) size, (b) concentration, and (c) zeta potential (ZP) were observed. However, while both systems show polydispersity index (PDI) less than 0.2, indicating a very homogeneous nanoparticle population (d), Lipo‐Dex have a lower PDI, as compared to Lipo. (e) Representative Cryo‐TEM images of Lipo and Lipo‐Dex verified that no structural changes occurred after encapsulating Dex within Lipo. Scale bar represents 100 nm for all the images. Lipo and Lipo‐Dex were stable in terms of (f) size, (g) ZP, and (h) PDI over a period of 28 days stored at 4°C. (i) Lipo‐Dex show a burst Dex release (~80%) after 4 h at 37°C. Results are shown as mean ± SEM. ****p < 0.0001. Cryo‐TEM, Cryo‐transmission electron microscopy.

Dex encapsulation had no effect on the average diameter (size), Lipo concentration, ZP, and PDI (Figure 2a–d). Interestingly Lipo‐Dex has a lower PDI, as compared to Lipo; but both are below 0.2, which is an acceptable value and indicates a homogenous population of phospholipid nanoparticles.^29^ Lipo and Lipo‐Dex showed an average size of 129.1 ± 3.4 and 115.9 ± 4.8 nm, PDI of 0.12 ± 0.01 and 0.04 ± 0.001 a.u., ZP of −10.0 ± 0.3 and − 9.4 ± 0.1 mV, and Lipo concentration of 1.78E+13 ± 0.17 and 1.94E+13 ± 0.13 (particles/mL), respectively (*n* = 3). Further, Lipo‐Dex was incubated with 10% FBS to evaluate how the adsorption of factors from the serum and ionic strength/osmotic pressure affect physicochemical parameters of the system (Table S1). Similar to previous reports,^30^ the incubation with serum yielded in a decrease in the Lipo‐Dex size to 101.8 ± 11.9 nm and an increase in PDI, indicating a decrease in homogeneity (PDI 0.30 ± 0.03). There was also a slight change in the system ZP to −5.1 ± 1.0 mV. Cryo‐TEM images of both Lipo and Lipo‐Dex (Figure 2e) demonstrated a similar bilayer structure and morphology in both formulations resulting in an average size of 9.4 ± 0.1 and 8.7 ± 0.2 nm for Lipo and Lipo‐Dex, respectively. The thickness of the lipid bilayer was measured for 10 Lipo using the Fiji software. Furthermore, the structural stability assessment at 4°C, followed by DLS measurements for Lipo and Lipo‐Dex size, PDI, and ZP, showed no changes in the liposomal physicochemical characteristics over the 28‐day period (Figure 2f–i). The release profile of Dex was assessed over 72 h at 37°C (Figure 2i). We determined that over 80% of encapsulated Dex to be released after 4 h from Lipo‐Dex according to our previous work.^31^ The Dex release profile of Lipo stored at 4°C at the following post‐synthesis points: 0, 7, 14, 21, and 28 days was evaluated. Dex release from Lipo increased by 41.6 ± 9.8%, 60.5 ± 8.8%, 71.4 ± 8.5%, and 78.8 ± 7.1% at the end of 7, 14, 21, and 28 days, respectively (*n* = 3). The concentration of encapsulated Dex was 1.5 ± 0.1 mg/mL (*n* = 3) with 51.2% Dex entrapment capacity (*n* = 3) and the mass loading of Dex was 100.3 ± 0.7 μg/mg lipid.

### 3.2 Biocompatibility and efficacy of Lipo‐Dex suppressing inflammation in neuronal cells

**FIGURE 3:**
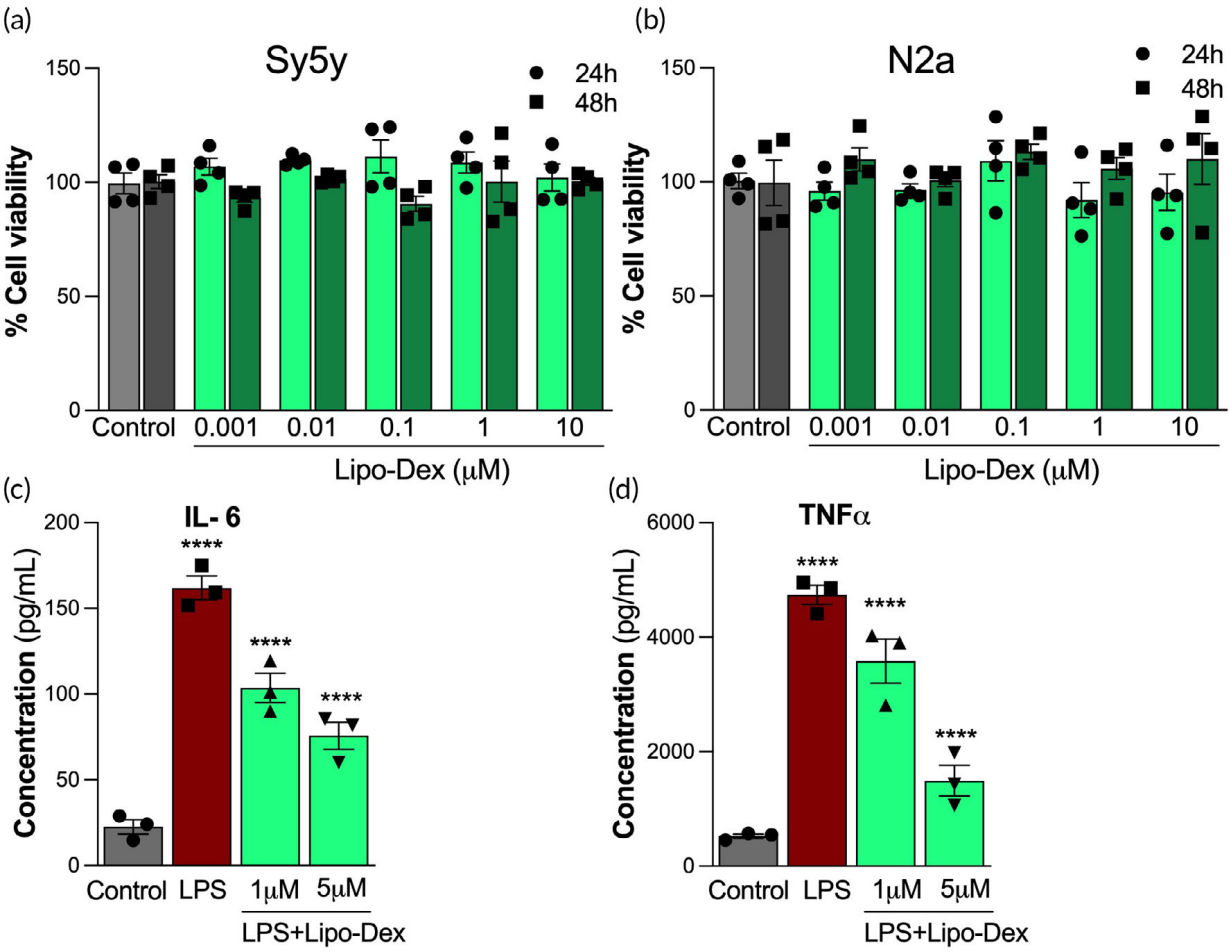
Lipo‐Dex is biocompatible and decreases inflammation in neuronal cells in vitro. In vitro biocompatibility of Lipo‐Dex in neuronal cells. Sy5y human neuronal cells (a) and N2a mouse neuronal cells (b) incubated with different concentrations of Dex delivered by Lipo‐Dex. Sy5y neuronal cell line pre‐treated with Dex (5 μM) delivered from Lipo‐Dex for 1 h before induction of inflammation with LPS (5 μg/mL) for 24 h. No significant differences were found between Lipo‐Dex treated and untreated cells control groups, indicating the biocompatibility of the system with neuronal cells. IL‐6 (c) and TNF‐α (d) levels were reduced in Sy5y cells inflamed with LPS and then treated with Lipo‐Dex for 24 h. Data are expressed as mean ± SEM from three independent experiments. ****p < 0.0001.

We first evaluated the biocompatibility of Lipo‐Dex in vitro using WST‐1 assay in Sy5y human neuronal cells and N2a murine neural cells and Lipo‐Dex were not well tolerated over Dex concentration range of 0.001–10 μM in both cell lines and did not show a significant difference in the cell viability compared to the untreated control at 24 and 48 h posttreatment (Figure 3a,b). Further, we tested the ability of Lipo‐Dex to suppress LPS‐induced inflammation in vitro. When administered at a concentration of 5 μg/mL, LPS significantly increased the expression levels of inflammatory cytokines IL‐6 and TNF‐α in Sy5y cells compared to those administrated with no LPS. Lipo‐Dex at a Dex concentration of 1 μM and 5 μM significantly suppressed the release of IL‐6 and TNF‐α (Figure 3c,d).

### 3.3 Lipo‐Dex biodistribution and organ accumulation in a mouse model of TBI

**FIGURE 4:**
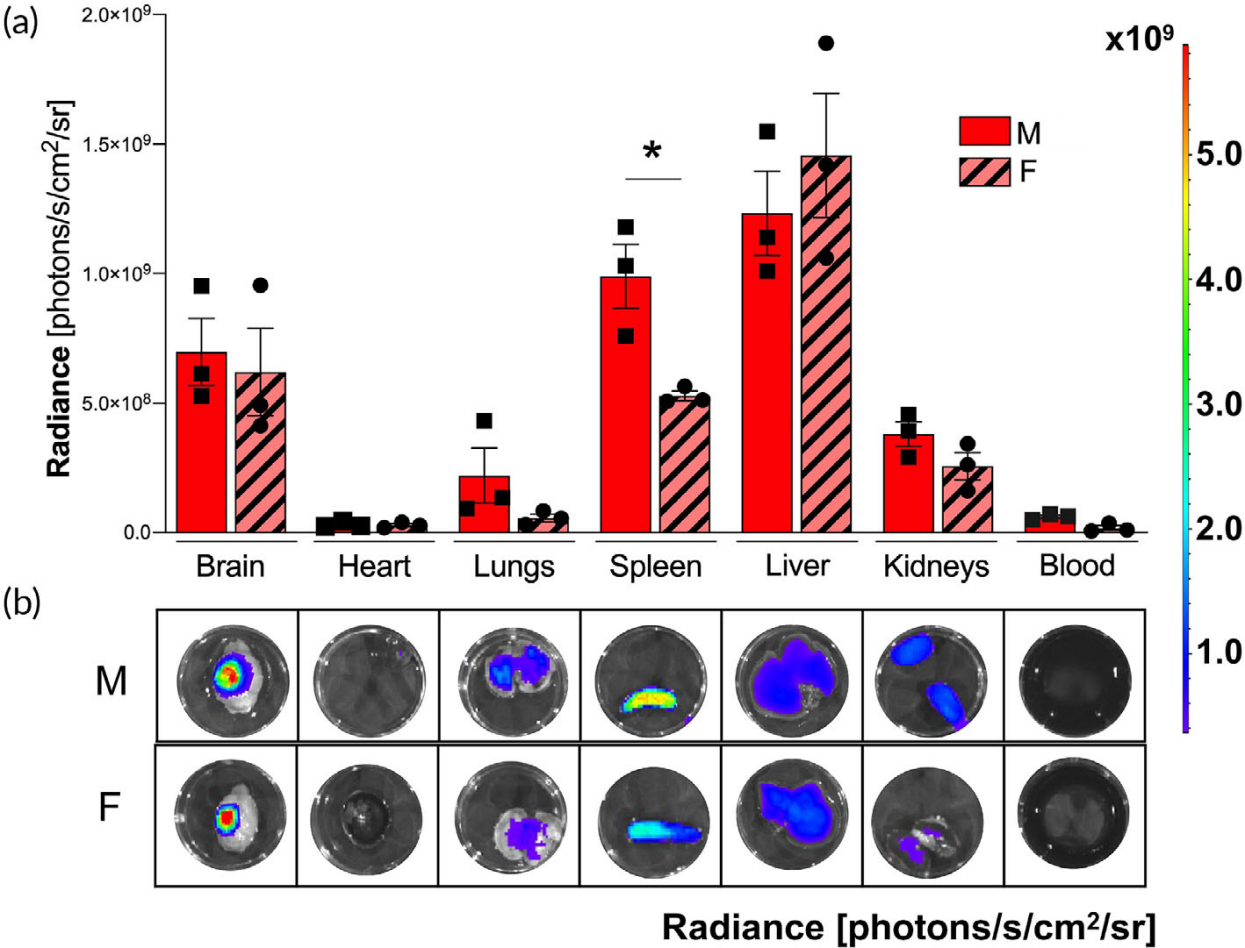
Sex‐dependent Lipo‐Dex biodistribution and brain targeting in vivo in TBI mouse model. Both male and female mice were administrated Cy5.5‐labeled Lipo‐Dex via retro‐orbital injection under anesthesia. (a) Lipo‐Dex demonstrated higher accumulation in the brain and filtering organs (spleen and liver). Interestingly, there was a significantly higher accumulation in the spleens of males compared to females. One‐way ANOVA followed by Tukey's multiple comparison test was used to determine statistical probabilities between brain biodistribution of Lipo‐Dex in male and female mice for heart, lungs, spleen, liver, kidneys, and blood. Results are shown as mean ± SEM. *p ≤ 0.05, *n* = 3/group. (b) Representative image of each organ (brain, heart, lungs, spleen, liver, and kidneys) and blood. TBI, traumatic brain injury.

We set out to characterize the effect of Lipo‐Dex in vivo in a mouse model of moderate TBI. Mice received intravenous administration of Lipo‐Dex 10 min after TBI. We evaluated the biodistribution of Lipo‐Dex at a dose of 3 mg Dex/kg by using Cy5.5‐labeled Lipo and in vivo imaging with IVIS. We found that Lipo‐Dex was present in several organs of the TBI mice (Figure 4a,b). Lipo‐Dex demonstrated significant accumulation in the brain at the site of the lesion, consistent with our previously reported data on the biodistribution of empty Lipo in TBI mice.^25^ Lipo‐Dex was also accumulated in the filtering organs, spleen and liver, lungs, and kidneys, but was not detected in the heart and blood. Male and female mice in the Lipo‐Dex group exhibited similar patterns of Lipo‐Dex accumulation in the brain lesions and other tissues except for spleen, showing a 47% increase in male mice compared to female mice (Figure 4a,b). Following this, the specified organs were retrieved, cleansed, fixed, sliced into sections, and subjected to hematoxylin and eosin (H&E) staining. This was done to assess whether both Lipo‐Dex and Lipo were well‐tolerated in terms of tissue damage in vivo. Upon comparison with tissues obtained from a control mouse injected with Lipo, no discernible abnormalities or pathological differences in morphology were detected between the two groups of organs (Figure S1).

### 3.4 Neuroprotective effects of Lipo‐Dex in vivo in TBI mouse model

**FIGURE 5:**
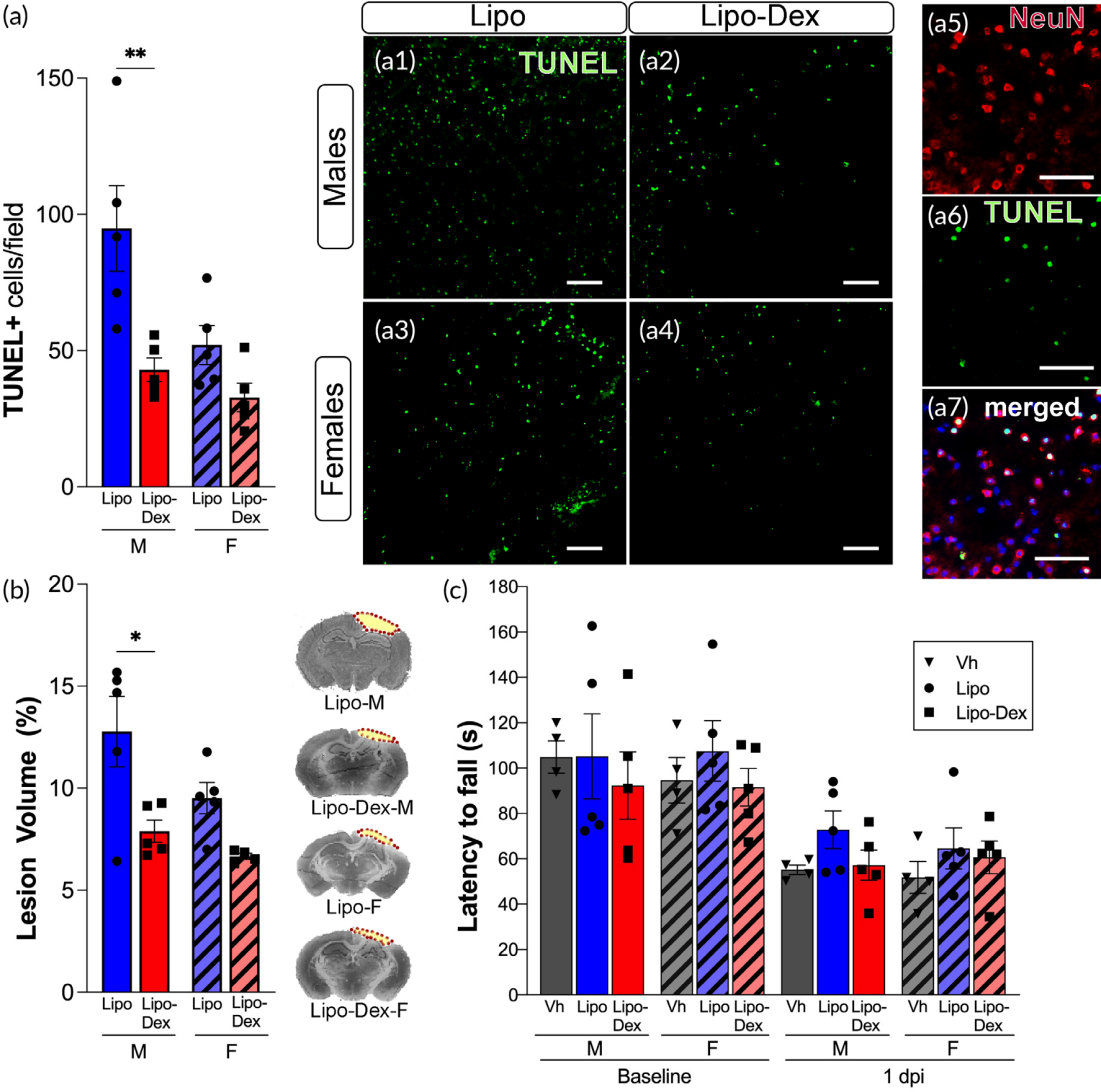
Lipo‐Dex treatment reduces neuronal death and lesion volume in males but does not improve motor ability after TBI. Lipo‐Dex administration reduces the number of TUNEL positive cells (green) (a, a1–a4) in males following TBI, colocalizing with positive neurons (NeuN, red) (a5–a7). Scale bar in a1–a4 represents 50 μm and a5–a7 represents 20 μm. Lesion volume is reduced by Lipo‐Dex treatment in males (b) as are shown the representative images of brain sections stained with cresyl‐violet at 1‐day post‐TBI. The dotted line indicates the lesion area composed of the cavity and edematous area. Lipo‐Dex‐treated mice did not improve motor recovery using the rotarod (c) compared to untreated neither Lipo groups. One‐way ANOVA followed by a Tukey's multiple comparison test was used and results are shown as mean ± SEM with *p ≤ 0.05, **p ≤ 0.01, *n* = 4‐5/group. TBI, traumatic brain injury.

To assess the acute neuroprotective effects of Lipo‐Dex after TBI, we quantified the dying cells in the pericontusional area and the lesion volume. The findings showed a significant reduction of 54% in TUNEL‐positive cells (Figure 5a, a1–a4). Upon examining the staining of TUNEL‐positive cells with the neuronal marker NeuN, we observed a high degree of colocalization in the cortical region indicating that most of the cells undergoing cell death are neurons (Figure 5a, a5–a7). We also found a reduction of 38% in lesion volume (Figure 5b) in male mice treated with Lipo‐Dex compared to those treated with Lipo. Interestingly, no significant differences were observed between the two groups in females. To evaluate the motor ability after Lipo‐Dex administration in TBI mice, a rotarod test was conducted 1 day post‐injury. There was a reduction in motor ability in all injured animals compared to baseline values, as expected. The findings indicated that there was no significant improvement in functional motor ability in either male or female mice treated with Lipo‐Dex compared to those treated with Lipo or untreated (Vh, vehicle) (Figure 5c).

### 3.5 Lipo‐Dex reduces the microglia and macrophage density and pro‐inflammatory cytokines in the brain, but not in the peripheral blood following TBI

**FIGURE 6:**
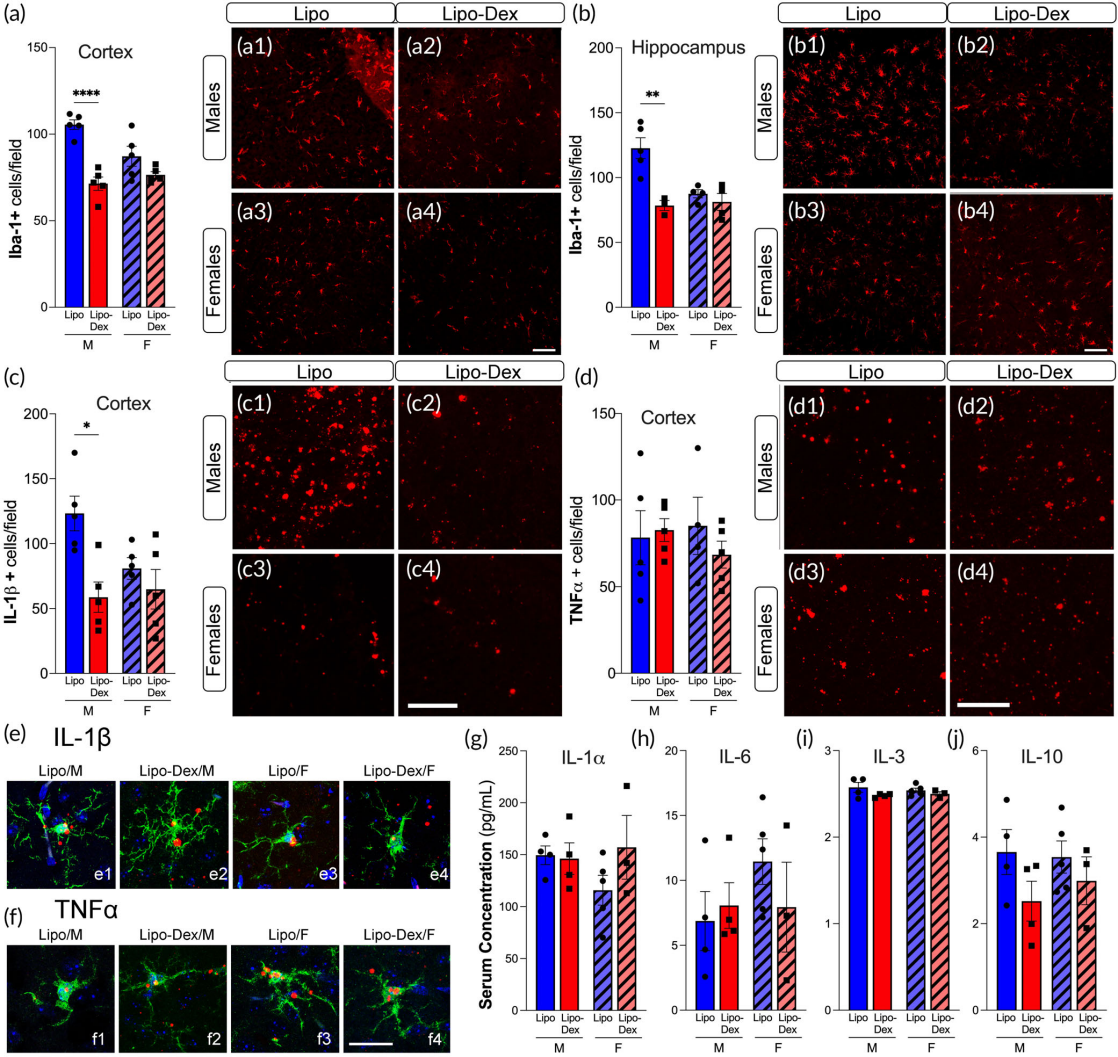
Lipo‐Dex reduces neuroinflammation following TBI. Microglia/macrophages (Iba‐1, red) positive cells decreased in males after TBI in the cortex (a, a1–a4) and hippocampus (b, b1–b4) compared to Lipo‐treated mice. The density of IL‐1β mRNA expression in the cortex (c, c1–c4) was reduced after Lipo‐Dex treatment, however we did not find changes in the TNF‐α mRNA expression (d, d1–d4). High‐magnification confocal images show the colocalization between Iba‐1 marker and IL1‐β (e) and TNF‐α (f) mRNA cytokine expression. The levels of pro‐inflammatory cytokines IL‐1 α (g), IL‐6 (h), IL‐3 (i), and anti‐inflammatory IL‐10 (j) are not altered after Lipo‐Dex treatment in serum blood samples. Scale bar represents 50 μm for a1–a4, b1–b4, c1–c4, and d1–d4, and 20 μm for e1–e4 and f1–f4. One‐way ANOVA followed by a Tukey's multiple comparison test was used and results are shown as mean ± SEM. *p < 0.05; **p < 0.01; ****p < 0.0001, *n* = 4–5/group. TBI, traumatic brain injury.

We evaluated the effect of Lipo‐Dex on the acute inflammatory response characteristic of TBI brains, including microglia/macrophage infiltration and release of pro‐inflammatory cytokines. In this quantitative analysis, our attention was directed toward the primary somatosensory cortex area situated proximate to the injury site's periphery. Following TBI, there was a rapid increase in the number and percentage of Iba‐1 positive cells in the injured cortex and hippocampus, which was significantly reduced in the Lipo‐Dex group at 1 day post‐TBI in male mice but not in females (Figure 6a, a1–a4, and Figure 6b, b1–b4). We further investigated the pro‐inflammatory response by measuring the mRNA expression of the cytokines TNF‐α and IL1‐β in injured brain sections using FISH. We observed a trend toward a reduced IL1‐β (Figure 6c, c1–c4) and TNF‐α (Figure 6d, d1–d4) expression in the ipsilateral peri‐contusion region in Lipo‐Dex‐treated males compared to Lipo‐treated males, but not in females. There was no detectable IL1‐β and TNF‐α mRNA expression in the contralateral hemisphere or sham brains (data not shown). TNF‐α and IL1‐β mRNA expression combined with Iba‐1 antibody using FISH demonstrated that most of the cytokine signal colocalized with microglia/macrophages markers (Figure 6e, e1–e4, f, f1–f4). We also assessed the changes in pro‐inflammatory and anti‐inflammatory cytokines in the plasma to determine if there is a systemic anti‐inflammatory effect following Lipo‐Dex administration. Dex administrated intraperitoneally 1 h post‐TBI significantly reduced the expression of pro‐inflammatory cytokines such as IL1‐β, TNF‐α, and IL‐6 in the brain, indicating its potential as an anti‐inflammatory agent.^32^ Our data showed no differences in serum concentrations of IL1α, IL‐6, IL‐3, or IL‐10 between Lipo and Lipo‐Dex‐treated mice, in either in males, or females, at 1 day post‐TBI (Figure 6g–j).

### 3.6 Lipo‐Dex treatment reduces astrogliosis following TBI

**FIGURE 7:**
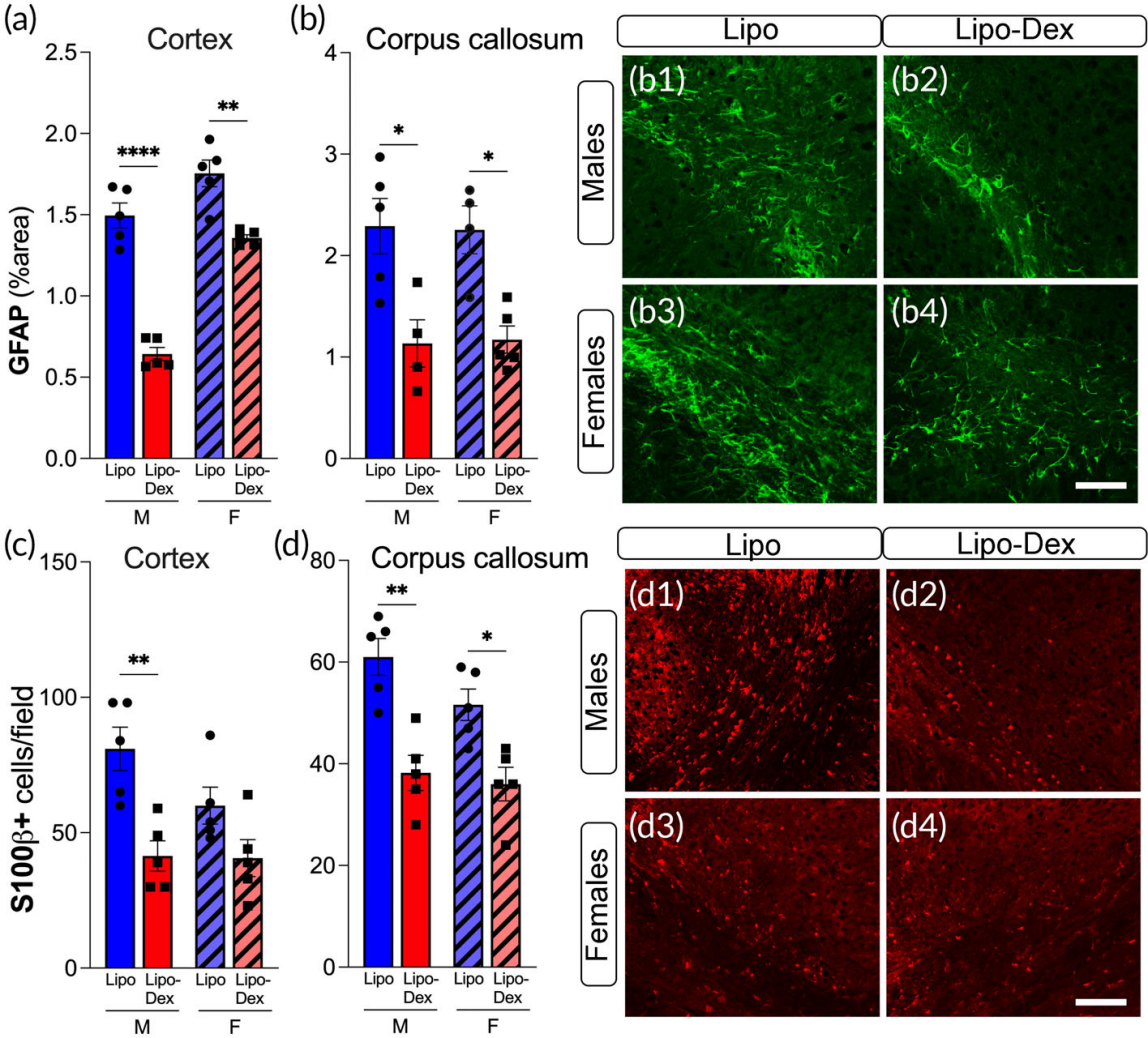
Lipo‐Dex treatment reduces astrogliosis after TBI. Astrogliosis (GFAP positive cells, green) decreases after Dex treatment in the cortex (a) and corpus callosum (b, b1–b4) in both males and females at 1‐day post‐TBI. Astrocytic density marked with S100β was decreased in Lipo‐Dex‐treated mice compared to Lipo‐treated after TBI in the cortex (c) in males and in the corpus callosum in males and females (d, d1–d4). Scale bar represents 50 μm. One‐way ANOVA followed by a Tukey's multiple comparison test was used and results are shown as mean ± SEM. *p < 0.05; **p < 0.01; ****p < 0.0001, *n* = 5/group. TBI, traumatic brain injury.

To determine whether Lipo‐Dex treatment altered the activation or survival of astroglial cells, we examined staining for glial‐specific markers in brain sections taken from Lipo or Lipo‐Dex treated mice at 1‐day post‐TBI. We used GFAP and S100β immunohistochemistry to assess the temporal and spatial distribution of astrogliosis and astrocyte density in male and female brains following TBI. Lipo‐Dex treatment significantly decreased the number of S100β‐positive cells and the amount of GFAP immunoreactivity in males and female brains at 1‐day post‐TBI. Lipo‐Dex administration also led to a change in the area occupied by the astrocytes in the cortex (Figure 7a,b, b1–b4) and reduced the number of astrocytes that were S100β‐positive in the cortex and corpus callosum at 1‐day post‐TBI (Figure 7c,d, d1–d4).

## 4 DISCUSSION

Dex is commonly prescribed to address cerebral edema and inflammation associated with TBI.^33^ However, its widespread systemic distribution, variable pharmacokinetic profile, and notable side effects have constrained its application. Considering these limitations, we expect that employing Lipo encapsulating Dex could offer an efficient remedy for mitigating TBI‐induced neuroinflammation and its related symptoms. This approach is poised to enhance the drug's delivery efficacy to the injured brain while preventing toxic effects. We anticipate that Lipo‐Dex could represent an effective solution for alleviating TBI‐induced neuroinflammation and its associated symptoms. This approach is expected to enhance the drug's delivery efficiency to the damaged brain and mitigate potential toxic effects.

In this study, we formulated a liposomal nanocarrier containing Dex. Our results revealed that Lipo‐Dex effectively decreased the release of inflammatory cytokines IL‐6 and TNF‐α in both human and murine neural cells. Our in vivo experiments demonstrated the targeted action of Lipo‐Dex on the injured brain, resulting in diminished lesion volume, reduced cell death, less astrogliosis, decreased pro‐inflammatory cytokine release, and attenuated microglial activation. Notably, these effects were more pronounced in male mice. Therefore, our findings emphasize the critical importance of considering gender as a significant factor in the development and assessment of nano‐therapies for brain injuries.

Previous studies have demonstrated the importance of consistent physiochemical properties in lipid‐based nanoparticle formulations for drug delivery. For example, the variations in the size and surface charge of lipid nanoparticles can significantly affect their cellular uptake and efficacy.^34^ Similarly, it was demonstrated that the stability of lipid‐based nanoparticles can be influenced by their physiochemical properties, which in turn can affect drug release and cellular uptake.^35^ Our results in the Lipo‐Dex fabrication are consistent with previous findings for lipid‐based formulations^36^ and suggest the successful fabrication of Lipo and Lipo‐Dex systems using the same extrusion parameters with no significant differences in their physiochemical properties, as reported in studies on the release of other corticosteroids such as methylprednisolone and hydrocortisone.^37^

Previous studies have shown that Dex can prevent LPS‐induced inflammatory responses in neuronal cells.^38^ Another study with a different liposomal formulation of Dex demonstrated a reduction in TNF‐α and IL‐6 secretion in inflamed primary human macrophages.^24^ IL‐6 and TNF‐α are the major inflammatory factors in the CNS that mediate inflammation related to trauma.^39^, ^40^ Our findings show that while being biocompatible in non‐LPS treated cells, Lipo‐Dex were able to mitigate LPS‐induced inflammation reducing these pro‐inflammatory cytokines. We further aimed to confirm in vitro findings through in vivo experiments, utilizing a thoroughly characterized animal model of TBI in our laboratory.^28^, ^41^, ^42^ In our previous study, we administered Lipo and leukosomes in mice following TBI,^25^ identifying accumulation of nanoparticles in the brain, lungs, spleen, liver, and kidneys.^25^ In the current study, we also observed an accumulation of Lipo‐Dex in the brain, heart, lungs, spleen, liver, and kidneys. The biodistribution of nanoparticles can be attributed to opsonization,^43^ an immunological process that uses opsonins to tag foreign pathogens, facilitating their phagocytosis by resident macrophages.^44^ This leads to a significant nanoparticle accumulation in organs such as the spleen and liver, causing a nonselective distribution of nanotherapeutics to peripheral tissues.^44^ Our results in this study showed a higher accumulation of Lipo‐Dex in the spleens of the male group. This could be explained by a higher inflammation in the spleen observed in males after TBI compared to females. In a study using a diffuse brain injury mouse model, it was observed that male mice had 31% more neutrophils, as measured in the spleen, compared to female mice with TBI.^45^

In our previous study, we also observed variations in central and peripheral inflammation between sexes,^28^ showing the peak of the inflammatory response at 1 day after TBI is exclusive to males. One explanation could be that the accumulation of nanomaterials in the tissue can disrupt the homeostasis of reactive oxygen species, with the liver and spleen being the primary targets of this heightened oxidative stress,^46^ particularly in males following TBI.^47^ Contradictorily, a study demonstrated the accumulation of Dex administered intravenously in the spleen of female mice after TBI,^48^ making a direct comparison with males unfeasible.

The decision to use Lipo‐Dex is based on the earlier studies wherein others employed free Dex in the same mouse model of TBI at almost one order of magnitude higher dose showing no response. Wei et al. conducted experiments with a copolymer‐based Dex prodrug (P‐Dex) in TBI mice,^48^ comparing its effectiveness to free Dex at 28 mg/kg administered intravenously. Their results revealed the incapacity of free Dex to alleviate neuroinflammation decreasing the pro‐inflammatory cytokine expression following TBI.

Nevertheless, through systemic administration, Lipo‐Dex primarily targets the inflamed brain. Administering Lipo‐Dex via i.v. could be a viable approach for delivering the drug to the brain in effective doses. Our previous study found that empty Lipo effectively reached injured and inflamed brain areas,^25^ and in the present work we found that Lipo‐Dex at a dose of 3 mg/kg, nine times lower than in the previous work by Wei et al., reduces the inflammatory response and reduces the lesion volume in a TBI mouse model.^48^ Our study also revealed that sex affects the efficacy of Lipo‐Dex treatment, with only male mice showing a significant reduction in inflammation and lesion size. The brains of female mice exhibit greater endogenous antioxidant enzyme activity compared to their male counterparts,^49^, ^50^ and this could explain the protective brain response of females compared to males after TBI. Hence, the neuroprotective impact of Dex is specifically observed in males during the acute phase of TBI, given their relatively less robust antioxidant system compared to females. The potential differences in endogenous antioxidant enzyme activity between males and females may be influenced by a variety of factors, including hormonal differences, genetic factors, and the specific type and severity of the brain injury.^51^ These findings emphasize the importance of considering sex when developing and evaluating new therapies for TBI.

The results suggested that there was no notable enhancement in motor skills for either male or female mice following treatment with Lipo‐Dex when compared to those treated with Lipo alone. Previous research has demonstrated that Dex led to substantial improvements in motor function and cognitive behavior compared to untreated mice after 14 days post‐TBI.^52^ It is crucial to emphasize that our current study primarily concentrates on assessing the impact of Lipo‐Dex during the acute stage following TBI, and any disparities in motor function improvement might occur at later time points.

Prior research has demonstrated that the intravenous administration of Dex following TBI effectively reduces the activation of microglia at the 14‐day post‐TBI.^48^ Furthermore, the discrepancies observed between male and female mice underscore notable sex‐related variations in their reactions to antioxidant nanoparticle delivery, implying the possibility of deriving maximum advantages from targeted antioxidant activity within the injured brain.^53^ It was observed that female mice exhibited higher levels of endogenous antioxidant activity, which delayed the increase in post‐traumatic oxidative stress markers compared to their male counterparts after TBI. Furthermore, separate studies have indicated that Dex‐induced TNF‐α overexpression may impact astrocytic hypertrophy without influencing microgliosis, suggesting a potential role in neuronal function following TBI.^54^

Dex has demonstrated the ability to diminish the expression of immune response genes, such as MCP‐1/CCL2 and ICAM‐1, which subsequently revert to baseline levels.^55^ Furthermore, Dex regulates NF‐KappaB levels, which increase in brain tissue early after TBI and induce an inflammatory response that can cause secondary brain injury. By preventing this secondary injury caused by inflammatory cytokines, Dex appears to have a protective effect on the injured brain.^56^ This suggests that the anti‐inflammatory impact of Dex was confined to the regions of the brain that sustained injury and did not affect inflammation in the peripheral blood. While some studies have failed to demonstrate that Dex can prevent or ameliorate gliosis in a neurodegenerative mouse model,^57^ others have found positive effects of Dex in different disease models. For example, in an epilepsy model, Dex administration has been found to improve inflammation, prevent astrogliosis (based on GFAP and S100β expression), and partially reduce astroglial dysfunction.^58^ Dex significantly reduced the GFAP signal in transgenic mice that overexpress GFAP, as measured by bioluminescent imaging,^59^ and caused a significant reduction in the number and density of astrocytes in the hippocampus and corpus callosum of postnatal rats.^60^

## 5 CONCLUSIONS

Our investigation unveils the encapsulation of Dex in Lipo as a promising strategy for the efficient delivery of glucocorticoids to young adult male and female C57BL/6 mice promptly following TBI, with a notably enhanced effectiveness observed in males. This approach leverages the small size of Lipo, enabling them to access the brain vasculature through the bloodstream, which is particularly advantageous when the BBB is compromised by TBI. Our results showed that Lipo‐Dex accumulated in the injured brain, resulting in reduced lesion volume, cell death, astrogliosis, release of pro‐inflammatory cytokines, and microglial activation compared to mice treated with Lipo alone. In addition, our in vitro studies demonstrated the favorable tolerance of Lipo‐Dex in human and murine neural cells. Furthermore, Lipo‐Dex exhibited significant suppression of inflammatory cytokines, IL‐6 and TNF‐α, after inducing neural inflammation with lipopolysaccharide. Interestingly, the anti‐inflammatory effects *in vivo* were found to be sex‐dependent, with a significant impact observed only in male mice, highlighting the importance of considering sex differences when administering this therapy, as male and female patients may exhibit differing responses to treatment. Taken together, our findings suggest that drug delivery methods, such as encapsulating drugs in Lipo, while also utilizing the effect of the carrier itself^25^ may offer new clinical opportunities for treating TBI and other neuropathological conditions. However, more research focusing on different lipid compositions and various physiochemical properties is necessary to optimize this approach and better understand the mechanisms underlying the observed sex‐dependent responses, and explore the potential for motor ability improvement at later time points. Further research and clinical trials are warranted to explore the translational potential of Lipo‐Dex in human TBI patients and to fully elucidate the effects of Dex, and the delivery carrier for improving brain function and recovery after TBI.

## AUTHOR CONTRIBUTIONS

**Gherardo Baudo:** Conceptualization (equal); data curation (equal); formal analysis (equal); investigation (equal); methodology (equal); writing – review and editing (supporting). **Hannah Flinn:** Formal analysis (equal); investigation (equal); methodology (equal); writing – review and editing (equal). **Morgan Holcomb:** Formal analysis (equal); investigation (equal); methodology (equal); writing – review and editing (equal). **Anjana Tiwari:** Formal analysis (equal); investigation (equal); methodology (equal); writing – review and editing (equal). **Sirena Soriano:** Investigation (equal); methodology (equal); writing – review and editing (equal). **Francesca Taraballi:** Conceptualization (supporting); investigation (supporting); writing – review and editing (supporting). **Biana Godin:** Conceptualization (equal); data curation (equal); formal analysis (equal); funding acquisition (lead); investigation (lead); methodology (equal); writing – review and editing (equal). **Assaf Zinger:** Conceptualization (lead); data curation (equal); investigation (equal); methodology (equal); supervision (equal); writing – review and editing (equal). **Sonia Villapol:** Conceptualization (lead); data curation (lead); formal analysis (equal); funding acquisition (lead); investigation (lead); methodology (equal); project administration (equal); resources (lead); supervision (lead); writing – original draft (lead); writing – review and editing (lead).

## FUNDING INFORMATION

This work was supported by grants from the National Institute of Neurological Disorders and Stroke (NINDS), R21 NS127265 (S.V., B.G.) and The Institute for Rehabilitation and Research (TIRR) through Mission Connect grant (S.V.). G.B. received funding from the Alliance of International Science Organizations (ANSO) Scholarship for Young Talents, the University of Chinese Academy of Sciences, the College of Material Science, and Opto‐electronic Technology.

## CONFLICT OF INTEREST STATEMENT

The authors declare no conflicts of interest.

## Supporting information


**Figure S1.** Lipo and Lipo‐Dex toxicity assessment in filtering organs. Tissue sections of spleen (a‐d), kidney (e‐h), lung (i‐l), and liver (m‐p) underwent hematoxylin and eosin (H&E) staining 1‐day post‐TBI in males and females. There were no pathological changes noted in the tissues between the groups treated with Lipo and those treated with Lipo‐Dex. Scale bar represents 50 μm.

**Table S1.** Effect of fetal bovine serum (FBS) on the physicochemical characteristics of Lipo‐Dex.

## Data Availability

The data that support the findings of this study are available from the corresponding author upon reasonable request.
