# Supplementary figures and images for: Sex‐dependent improvement in traumatic brain injury outcomes after liposomal delivery of dexamethasone in mice
Source: Bioeng Transl Med. 2024 Feb 4;9(4):e10647. doi: 10.1002/btm2.10647 (PMC11256133; doi:10.1002/btm2.10647)

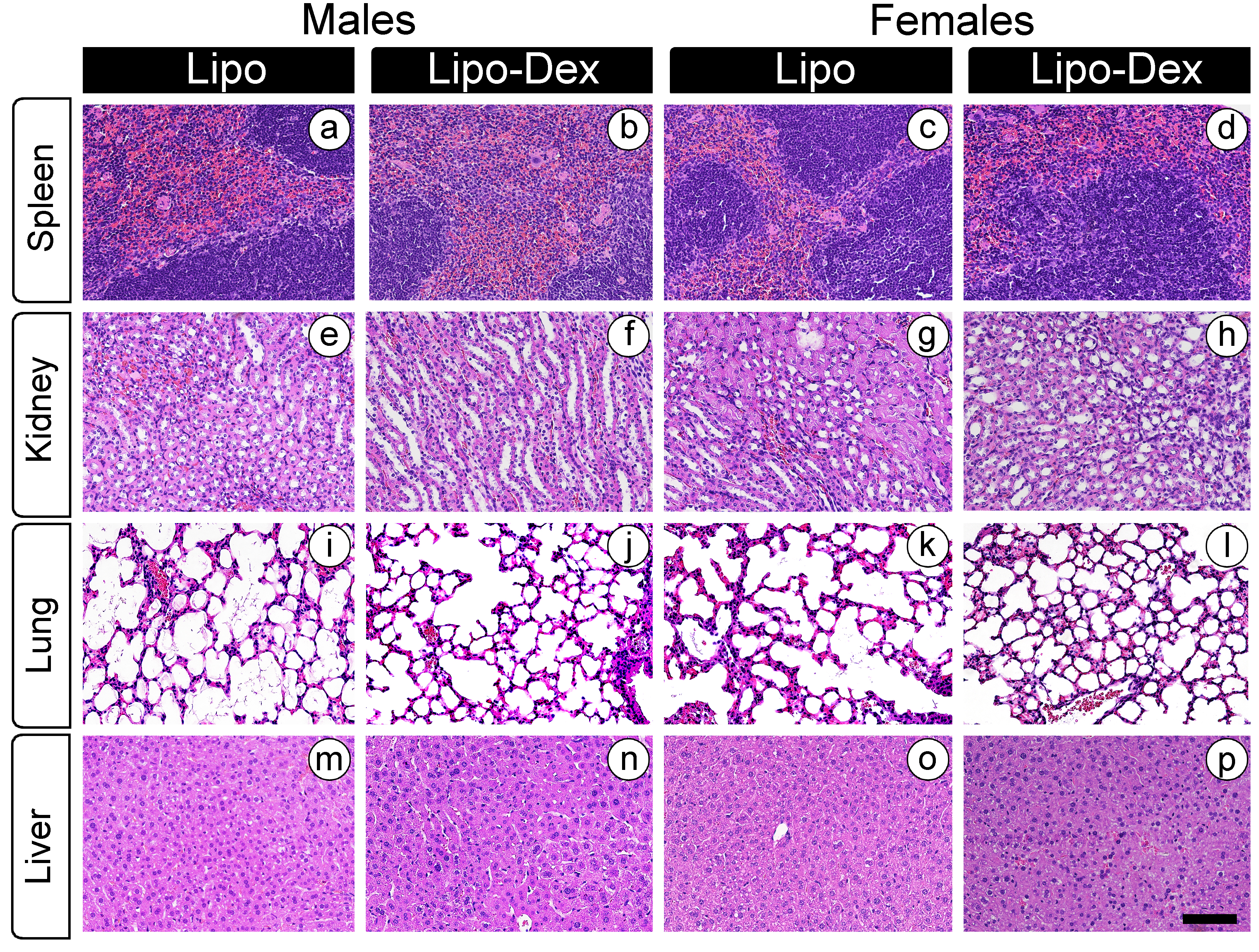

Supplement: Supplementary file 1 — Figure S1. Lipo and Lipo‐Dex toxicity assessment in filtering organs. Tissue sections of spleen (a‐d), kidney (e‐h), lung (i‐l), and liver (m‐p) underwent hematoxylin and eosin (H&E) staining 1‐day post‐TBI in males and females. There were no pathological changes noted in the tissues between the groups treated with Lipo and those treated with Lipo‐Dex. Scale bar represents 50 μm. [file BTM2-9-e10647-s001.jpg]
