# Supplementary material for: Sex‐dependent improvement in traumatic brain injury outcomes after liposomal delivery of dexamethasone in mice
Source: Bioeng Transl Med. 2024 Feb 4;9(4):e10647. doi: 10.1002/btm2.10647 (PMC11256133; doi:10.1002/btm2.10647)
Supplement: Supplementary file 2 — Table S1. Effect of fetal bovine serum (FBS) on the physicochemical characteristics of Lipo‐Dex. [file BTM2-9-e10647-s002.docx]

**Supplementary Table 1.** Effect of Fetal bovine serum (FBS) on the physicochemical characteristics of Lipo-Dex.

|  | 10% FBS | PBS |
| --- | --- | --- |
| Size [nm] | 101.8 ± 11.9 | 118.8 ± 3.7 |
| PDI [a.u.] | 0.30 ± 0.03 | 0.08 ± 0.03 |
| Zeta Potential [mV] | -5.1 ± 1.0 | -8.2 ± 0.7 |
